# SVAw - a web-based application tool for automated surrogate variable analysis of gene expression studies

**DOI:** 10.1186/1751-0473-8-8

**Published:** 2013-03-11

**Authors:** Mehdi Pirooznia, Fayaz Seifuddin, Fernando S Goes, Jeffrey T Leek, Peter P Zandi

**Affiliations:** 1 Department of Psychiatry and Behavioral Sciences, Johns Hopkins School of Medicine, Baltimore, MD, USA; 2 Department of Biostatistics, Johns Hopkins Bloomberg School of Public Health, Baltimore, MD, USA; 3 Department of Mental Health, Johns Hopkins Bloomberg School of Public Health, Baltimore, MD, USA

## Abstract

**Background:**

Surrogate variable analysis (SVA) is a powerful method to identify, estimate, and utilize the components of gene expression heterogeneity due to unknown and/or unmeasured technical, genetic, environmental, or demographic factors. These sources of heterogeneity are common in gene expression studies, and failing to incorporate them into the analysis can obscure results. Using SVA increases the biological accuracy and reproducibility of gene expression studies by identifying these sources of heterogeneity and correctly accounting for them in the analysis.

**Results:**

Here we have developed a web application called SVAw (Surrogate variable analysis Web app) that provides a user friendly interface for SVA analyses of genome-wide expression studies. The software has been developed based on open source bioconductor SVA package. In our software, we have extended the SVA program functionality in three aspects: (i) the SVAw performs a fully automated and user friendly analysis workflow; (ii) It calculates probe/gene Statistics for both pre and post SVA analysis and provides a table of results for the regression of gene expression on the primary variable of interest before and after correcting for surrogate variables; and (iii) it generates a comprehensive report file, including graphical comparison of the outcome for the user.

**Conclusions:**

SVAw is a web server freely accessible solution for the surrogate variant analysis of high-throughput datasets and facilitates removing all unwanted and unknown sources of variation. It is freely available for use at http://psychiatry.igm.jhmi.edu/sva. The executable packages for both web and standalone application and the instruction for installation can be downloaded from our web site.

## Introduction

Accurate analysis of high-throughput data in molecular biology is complicated because of the presence of underlying factors that cause unwanted heterogeneity in the data. An inherent issue with microarray expression studies is that of artefactual or confounded variation. In addition to the biological factors that are of interest to the researcher, gene expression levels are affected by other factors that are both technical and biological. A typical example is a batch effect, which can occur when some samples are processed differently than others [1]. These heterogeneity factors, also commonly known as batch effect, can range from genetic and environmental variables to demographic factors [2,3]. The impact of these factors may vary from a deviation in the outcome to potentially completely compromising the statistical or biological validity of a study [4]. The sva method developed by Leek *et al.*[4,5] addresses this issue by identifying, estimating and removing unwanted sources of variation in high-throughput experiments. The sva function estimates surrogate variables, which are covariates constructed directly from high-dimensional data, and then uses these variables in downstream analyses such as differential expression analysis. Here we employed the Leek *et al.* sva algorithm [4,5] and implemented a web application, called SVAw, to perform fully automated analysis in a user friendly web browser view. SVAw can also be downloaded as a fully functional automated standalone application.

SVAw is a tool that enables researchers to utilize Surrogate Variable Analysis when analyzing high throughput genomic data to capture such heterogeneities in the dataset that can potentially lead to biased analysis of the data. Additionally, the web server makes the Surrogate Variable Analysis more accessible to researchers with no programming background in order to identify and control for potential heterogeneity in their genomics data. The SVAw calculates probe/gene statistics such as the fold change and p-value for both pre (unadjusted) and post sva analysis (adjusted with sva) and generates a comprehensive report, including graphical comparison of the outcome.

## Results

A typical gene expression analysis involves identifying differential expressed genes among various experimental conditions. Statistical analysis will usually calculate the magnitude of fold changes as well as measures of statistical significance (p-values). SVAw generates pre- and post-Surrogate variable Analysis p-values and fold changes of the analyzed data. The statistical and graphical visualization of the data before and after the SVA procedure gives the user a clear sense of the extent of heterogeneity in their dataset. In particular, visualization of the data using three different graphical representations: 1) volcano plot; 2) MA plot and 3) histogram of p-values, draws the user’s attention to the different aspects of statistical analysis. The SVAw architecture simplifies the analysis of gene expression data by executing a computational workflow on a set of data uploaded by user. For a midsize dataset (50 samples) SVAw can process up to 1mb of raw data per minute using both the web and standalone applications on a quad core AMD processor with 8GB memory. The SVAw web server is currently able to process up to a 16MB input file size.

1. Data format

1.1. Input data: For SVAw, there are two required input files from the user: Expression and Factors file. The expression file is a *m x n* expression matrix with *n* arrays for *m* genes/probesets/probes. Factors file is a *m x 2* matrix with *m* individuals/arrays divided into groups. Column1 is the unique identifier (ID) and column2 is the group status as 1 stand for “Group1 arrays” (for e.g. "cases") and 0 for “Group2” arrays (for e.g. "controls").

1.2. Output: SVAw has a comprehensive output. All text files will be in plain-text in tabular format with each row terminated by a new-line character. These files can be easily used in spreadsheet applications and database programs (such as SQL databases) for further analysis. Output files include:

1.1.1. Surrogate_Variable.txt: An *m x n* matrix with m individuals/arrays and n surrogate variables predicted for the data set by SVAw.

1.1.2. Probe_Statistics.txt: A table of results for regression of gene expression on the primary variable of interest before and after correcting for surrogate variables. This file includes the following columns (fields) for each gene/probesets/probes:

[pre-sva] calculations:

Coefficient unadjusted: linear regression coefficient from the fitted model before sva adjustment.

Fold change: up or down regulated (unadjusted), log2 ratio of mean expression of Group1 (e.g., "cases") vs. Group2 (e.g., "controls") from the fitted model before sva adjustment.

p_unadjusted: p-value for the test of significance comparing the mean expression levels between groups (e.g., Group1 or “cases” vs. Group2 or “controls”) for all genes/probesets/probes from the fitted model before sva adjustment.

[post-sva] calculations:

surrogate variables included as "covariates" in the linear regression model of gene expression on the primary variable of interest:

Coefficient after sva adjusted.

Fold change: up or down regulated after adjustment with sva

p_adjusted: p-value for the test of significance after sva adjustment

1.1.3. Probe_Statistics_Significants.txt: A subset of the Probe_Statistics.txt table (above) of results by significance (p-value [post-sva] < 0.05). All genes/probesets/probes meeting this criterion are shown in this table.

**Figure 1:**
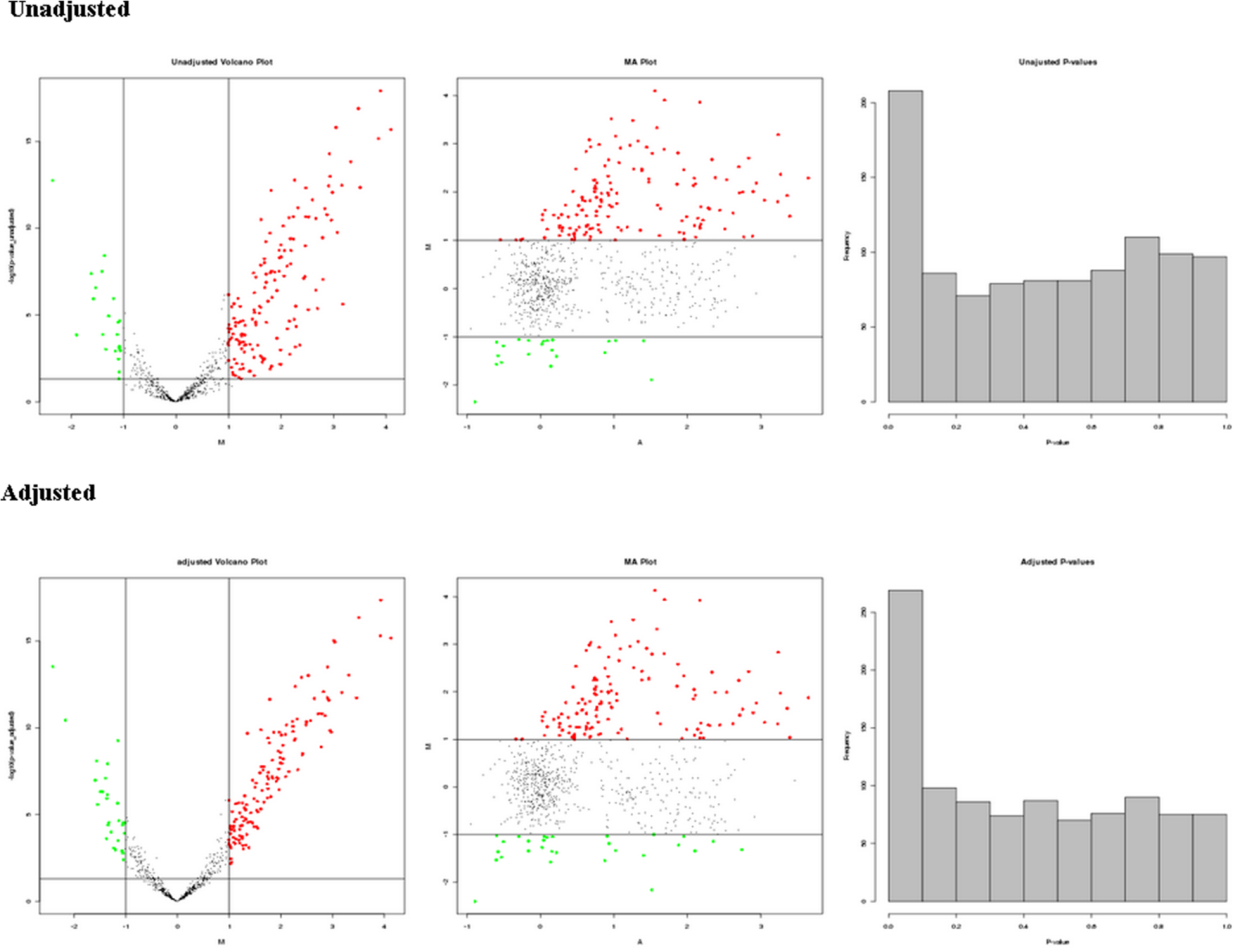
**Plot of SVA unadjusted and adjusted.** Left panel indicates volcano plot comparing the size of the fold change to the statistical significance level. Middle panel shows MA plot comparing the intensity ratio (M) and the average intensity (A). Right panel represents histogram of P-value from differential expression analysis.

1.1.4. Unadjusted and Adjusted Graph (Figure 1) files in PNG format: These files contains three plots:

▪ Volcano Plot: Plot to compare the size of the fold change to the statistical significance level. The x axis is the fold change between the two groups (on a log scale) indicating biological impact of the change, and the y axis represents the p-value for a *t*-test of differences between samples (on a negative log scale) indicating the statistical evidence, or reliability of the change.

▪ MA Plot: M (the intensity ratio) vs. A (the average intensity)

▪ Histogram: Histograms of the unadjusted and the sva adjusted p-values from differential expression analysis

1.2.5. **Report.htm**: A brief report of the analysis in html format.

2. Sample Tests

We tested the functionality of SVAw using well-known datasets from gene expression experiments with different sizes of inputs, as well as simulated datasets. Some of the datasets include genome-wide expression studies using autopsy brain samples from the Stanley Medical Research Institute (SMRI) comparing bipolar and depression cases and controls. Details of the methods and results are provided online at Metamoodics web resource [6].

## Methods

1. SVA

We employed Surrogate variable estimation based on the algorithms in Leek and Storey [4,7]. The method has been described before. Briefly, the SVA algorithm can be performed in four steps: 1) Removing the signal due to the primary variables of interest and obtain a residual expression matrix and identify signatures of heterogeneity, 2) Identifying the subset of genes that are driving each signature of expression heterogeneity, 3) Building a surrogate variable for each subset of genes, and 4) Including all significant surrogate variables as covariates in regression analyses. Surrogate variables are estimated using either the iteratively re-weighted (IRW) or the two-step surrogate variable analysis algorithm [8]. The basic idea of IRW is to construct a specified number of surrogate variables from a gene expression data set and a fixed model. The two steps function, on the other hand, estimates surrogate variables based on the subset of rows affected by unmodeled dependence [3,9]. The default method in SVAw is IRW.

2. Univariate linear regression

Univariate linear regression [10] analysis is first applied and linear regression coefficients are obtained from the fitted models. We then calculate the log2 ratio (Fold Change (FC)) of mean expression levels for group1 vs. group2 from the fitted model before sva adjustment. P-values for the test of significance for each genes/probesets/probes comparing mean expression levels between the two groups is also estimated from the fitted model before sva adjustment.

3. Multivariate linear regression

We incorporate the surrogate variables as cofounders and perform multivariate linear regression [10]. FCs and p-values for each genes/probesets/probe are then calculated from the fitted models after the sva adjustment.

## Implementation

1. Web Server System Architecture

**Figure 2:**
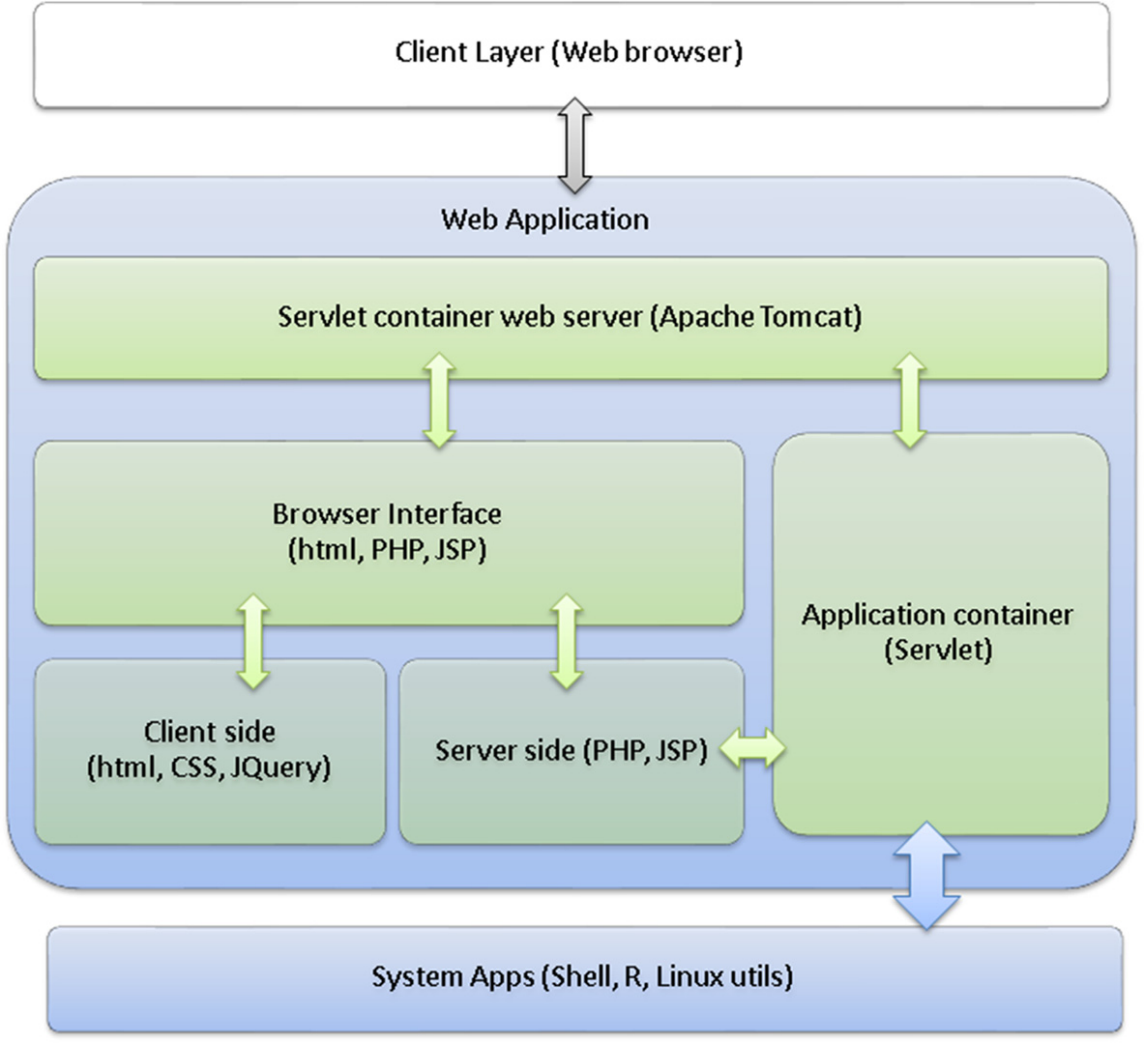
**Web server 2-tier client server system architecture.** Flow chart demonstrates the components of the servlet container web server (Tomcat) communicating with system applications such as shell utils and R packages to perform SVA analysis, univariate and multivariate regression.

**Figure 3:**
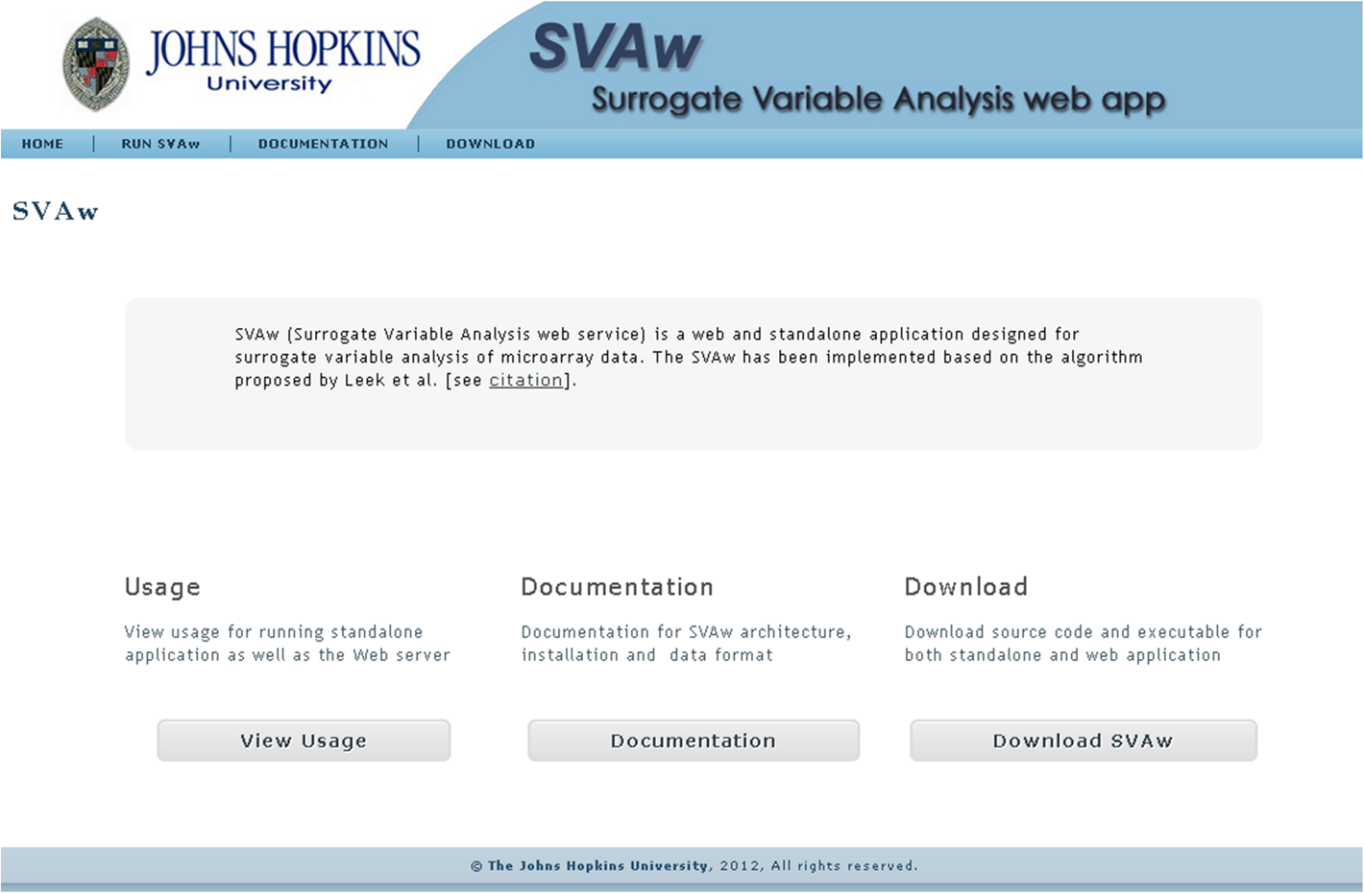
**Screenshot of the SVAw web server**. The analysis can be performed through the website or by downloading the standalone application. The website also provides details on installation and usage.

The Web Server is designed and implemented on the basis of two-tier client–server architecture; a presentation layer or front-end and a business layer or back-end. Figure 2 illustrates the core components of the system. Java Servlet container Apache's Jakarta Tomcat application server [11] has been employed to take advantage of state-of-the-art Java technologies [12] in order to provide effective client server-side application execution. The front-end web application program (Screenshot shown in Figure 3), constructed based on JQuery [13], and Java Server Page (JSP) 2.0 technology [14], is in charge of task submissions and results displays. The back-end program consists of series of servlets [15] that communicates with system resources and utilities. Several open source applications and technologies [16-19] have been employed to enhance the flexibility and extendibility of this component. Open source R statistical analysis packages [18,19] are mainly in charge of the data analysis and processing. The web application is hosted on Apache Tomcat web server [20] on a Red Hat Linux operating system [21]. A war file (Web application ARchive) [22] format can be downloaded from our website and imported to any Servlet container web server.

1.1. Download and install the pre-requirements:

Download and install R package [18].

Download and install “corpcor” R package [23]:

wget http://cran.r-project.org/src/contrib/corpcor_1.6.4.tar.gz

R CMD INSTALL corpcor_1.6.2.tar.gz

Download and install “qvalue” R package [24]:

wget http://www.bioconductor.org/packages/2.9/bioc/src/contrib/qvalue_1.28.0.tar.gz

R CMD INSTALL qvalue_1.28.0.tar.gz

Download and install “corpcor” R package [5]:

wget http://www.bioconductor.org/packages/2.11/bioc/src/contrib/sva_3.4.0.tar.gz

R CMD INSTALL sva_3.1.2.tar.gz

1.2. Web application Installation:

Download and install TOMCAT [11].

Download SVAw.war

wget http://psychiatry.igm.jhmi.edu/sva/SVAw.war

Deploy war files into the webapps directory:

\Tomcat home\webapps

Start TOMCAT

SVAw.war is automatically extracted, then becomes available in:

http://127.0.0.1:8080/sva/

2. Standalone Application

2.1. Installation for the standalone application:

2.1.1. Download and install the pre-requirements:

This is the same as section 1.1 (pre-requirements for web application).

2.1.2. Download and install the application

Download and unzip the standalone application:

wget http://psychiatry.igm.jhmi.edu/sva/package/SVAw_standalone.tar.gz

tar -zxvf SVAw_standalone.tar.gz

The SVAw standalone executable package has been designed and implemented using Shell scripting language. The packages can be run on 32 and 64-bit Linux system. Before running the packages, some essential R packages declared in the download page are needed to be installed in the system.

## Conclusion

SVAw is a web-based freely accessible solution for the surrogate variant analysis of high- throughput datasets and should facilitate removing all unwanted and unknown sources of variation. It provides users to analyze gene expression data directly and simply by an easy-to-use browser user interface. Moreover, we also provide executable standalone packages for users to incorporate and build their pipeline locally.

## Availability and requirements

Project name: SVAw (Surrogate variable analysis Web app)

Project home page: http://psychiatry.igm.jhmi.edu/sva

Operating system(s): Platform independent

Programming language: Java 1.5.0 or higher, R v2.10 or higher

Other requirements: Tomcat 5.5 or higher, supported web browsers: Firefox3+, chrome, safari3+, Internet Explorer8+

License: GNU GPL

Any restrictions to use by non-academics: none

## Competing interests

The authors declare that they have no competing interests.

## Authors’ contributions

MP, JTK and PPZ conceived and designed the project. MP and FS wrote and ran the algorithms. MP constructed and designed the web-server and coded the web application. FS performed the experimental analysis. MP, FSG and PPZ directed to project. All authors read, contributed to and approved the final manuscript.
